# Thickness Dependence of Superconductivity in Layered Topological Superconductor *β*-PdBi_2_

**DOI:** 10.3390/nano11112826

**Published:** 2021-10-24

**Authors:** Huijie Li, Huanhuan Wang, Wenshuai Gao, Zheng Chen, Yuyan Han, Xiangde Zhu, Mingliang Tian

**Affiliations:** 1 Information Materials and Intelligent Sensing Laboratory of Anhui Province, Institutes of Physical Science and Information Technology, Anhui University, Hefei 230601, China; lihjlm@163.com (H.L.); 17368835892@163.com (H.W.); 2 Anhui Key Laboratory of Condensed Matter Physics at Extreme Conditions, High Magnetic Field Laboratory, HFIPS, Anhui, Chinese Academy of Sciences, Hefei 230031, China; chenz911@mail.ustc.edu.cn (Z.C.); yyhan@hmfl.ac.cn (Y.H.); xdzhu@hmfl.ac.cn (X.Z.); tianml@hmfl.ac.cn (M.T.); 3 Department of Physics, University of Science and Technology of China, Hefei 230031, China; 4 School of Physics and Optoelectronic Engineering, Anhui University, Hefei 230601, China

**Keywords:** topological superconductor, nanoflakes, PdBi_2_, transport property

## Abstract

We report a systematic study on the thickness-dependent superconductivity and transport properties in exfoliated layered topological superconductor *β*-PdBi_2_. The superconducting transition temperature *T*_c_ is found to decrease with the decreasing thickness. Below a critical thickness of 45 nm, the superconductivity is suppressed, but followed by an abrupt resistance jump near *T*_c_, which is in opposite to the behavior in a superconductor. We attribute suppressed *T*_c_ to the enhanced disorder as the thickness decreases. The possible physical mechanisms were discussed for the origination of sharply increased resistance in thinner *β*-PdBi_2_ samples.

## 1. Introduction

Topological superconductors are characterized by a full paring gap in the bulk and topologically protected gapless states that can support massless Majorana fermions [1]. These unique states make it attractive for applications in spintronics and quantum computation [2,3]. Such states can be achieved not only in a carrier dopped topological insulator but also in pure stoichiometric compound [4,5,6,7,8,9]. The recently discovered superconductor *β*-PdBi_2_ provides a promising candidate for the long-sought stoichiometric topological superconductor [10,11,12,13]. Previous studies have shown that *β*-PdBi_2_ holds a superconducting transition temperature, ranging from 4.25 K to 5.4 K, depending on sample quality [14,15,16]. Angle-resolved photoemission spectroscopy (ARPES) reveals the presence of topological surface states with in-plane spin polarizations [15], which is useful for future spintronics research using a topological superconductor. Furthermore, *β*-PdBi_2_ is of particular interest due to its naturally layered crystal structure, which presents the opportunity to understand how a gradual reduction in dimensionality affects its properties [17,18,19]. In previous studies, theoretical calculations verify that *β*-PdBi_2_ film could harbor topological surface states with layer dependence [20]. While single-layer *β*-PdBi_2_ was proposed to be a two-dimension superconductor with topological edge states [12,21]. These results demonstrate that *β*-PdBi_2_ may provide a reliable platform for achieving the long-sought-after topological superconductor in the low-dimensional limit. In addition, the superconductivity can be suppressed [22] or distinctly enhanced [23] and even undergoes a superconductor-insulator transitions [24] with decreasing thickness, which provides a crucial means of understanding the phase coherence of cooper pairs of two-dimensional superconductors. Several studies reported that 2*H*-NbSe_2_ exhibits suppressed superconductivity with decreasing thickness [25], while in 2*H*-TaS_2_ the superconductivity is enhanced [22]. Due to the thickness-dependent quantum size effects, the superconducting transition temperature displays oscillating behavior when the Pb film thickness increases layer by layer [26]. Up to now, the investigation of thickness dependence of transport properties in *β*-PdBi_2_ is still lacking. It is essential to investigate superconductivity in ultrathin *β*-PdBi_2_ nanoflakes and to detect the possible influence on its topological aspect.

In this study, we performed systematic transport research on *β*-PdBi_2_ nanoflakes with various thicknesses and found that the superconducting transition temperature is gradually suppressed with decreasing thickness, and finally vanishes when the thickness is down to 45 nm. Unexpectedly, when the thickness of the flake is below 36 nm, we observed an abrupt upturn in resistance near 7 K, followed by a plateau with further decreasing temperatures. When applied to a magnetic field, the onset temperature of the upturn resistance was pushed to low temperatures and can be completely suppressed with further increase in the field. The possible mechanism for these unusual properties was discussed in terms of the enhanced disorder as the thickness decreases.

## 2. Experimental Methods

**Figure 1:**
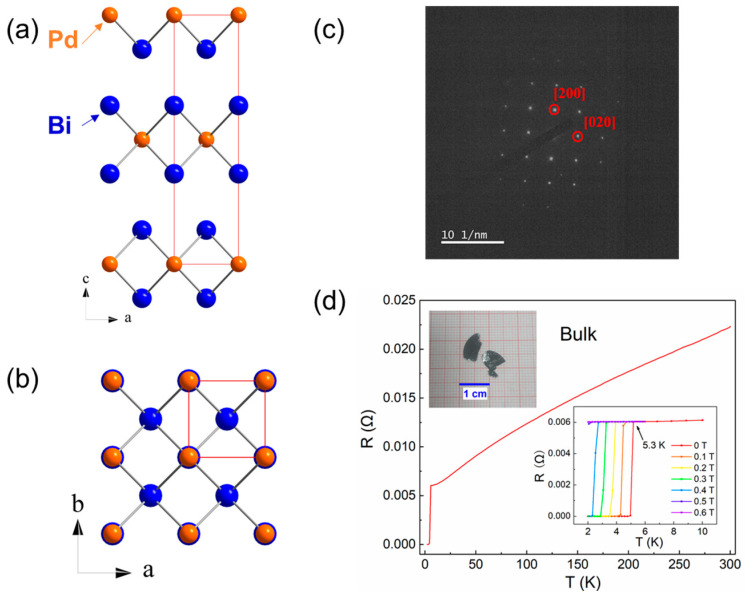
Crystal structure of *β*-PdBi_2_ in (**a**) side view and (**b**) top view. The red solid lines display the conventional body-centered tetragonal unit cells. (**c**) The selected area electron diffraction pattern of *β*-PdBi_2_. (**d**) Temperature dependence of resistance in bulk *β*-PdBi_2_. Inset is R-T curves at difference magnetic fields along *c* axis.

The *β*-PdBi_2_ single crystals were grown by a melt growth method, as described in [15]. The synthetic centimeter-scale *β*-PdBi_2_ crystals were platelike with silvery surfaces, as shown in the inset of Figure 1d. *β*-PdBi_2_ has a layered crystalline structure with the centrosymmetric space group *I*4*/mmm*. Each Pd atom was located at the center of eight Bi atoms, forming the layered unit cell, as shown in Figure 1a,b. The crystal bonding between the PdBi_2_ layers is van der Waals force in nature. The *β*-PdBi_2_ nano-devices with standard Hall-bar were fabricated using EBL technology. The *β*-PdBi_2_ flakes with various thickness were mechanical exfoliated from the bulk crystals and then transferred onto SiO_2_/Si substrates with a polydimethylsiloxane (PDMS) stamp. The desired flakes can be preliminary selected via shape and optical contrast under an optical microscope. The precise thickness values were identified through atomic force microscope (AFM) measurements. The standard six-electrode patterns covered with Ti/Au (5 nm/50 nm) were transferred to *β*-PdBi_2_ flakes by EBL technology followed by thermal evaporation process. The finished devices were covered with PMMA and further protected from water vapor and oxygen in the inert atmosphere glove box. Thickness identification of these flakes were carried out on atomic force microscope (NX10, Park Inc, Suwon, Korea). The Selected Area Electron Diffraction (SAED) experiments were performed on Talos F200X transmission electron microscope (TEM, Thermo Scientific Inc, Waltham, MA, USA). The EBL experiments were using the ultra-high resolution electron beam-lithography system (e-Line Plus, Raith Inc. Dortmund, Germany). Magnetotransport measurements were carried out using a 16 T physical property measurement system (PPMS, Quantum Design Inc, San Diego, CA, USA).

## 3. Results and Discussion

The SAED pattern in Figure 1c demonstrates clear tetragonal crystal orientation, which confirms the crystal structure of *β*-PdBi_2_. Figure 1d shows the temperature dependence of resistance, a sharp superconducting transition at *T_c_* = 5.3 K is observed. When applied to a magnetic field along the *c* axis, the superconductivity is strongly suppressed with a critical field of about *H*_c_ = 0.6 T, as shown in the inset of Figure 1d. These superconducting characteristic parameters are consistent with previous reports [15,27,28]. 

**Figure 2:**
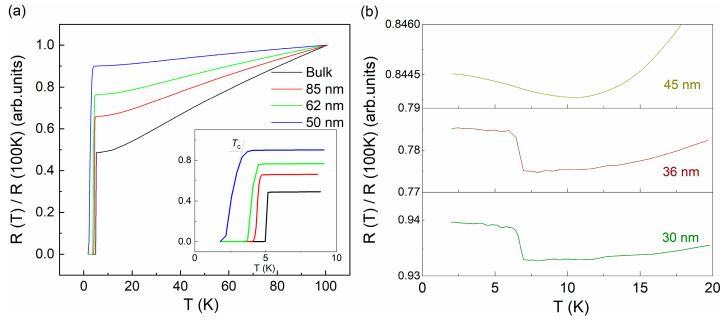
(**a**) and (**b**) Temperature dependence of normalized resistance, R(T)/R (100 K) for a series of *β*-PdBi_2_ nanoflakes with different thickness. The inset of (**a**) is a close-up of the curves near the *T*_c_.

To investigate the influence of size confinement on the transport properties of *β*-PdBi_2_, Figure 2a shows the normalized resistance versus temperature of nanoflakes with different thicknesses. The inset is a close-up of the curves in the range from 2 K to 10 K. Obviously, the superconducting transition temperature *T*_c_ decreases gradually when the thickness is reduced down to 50 nm, followed by a broadening of the transition. Strikingly, when the thickness is about 45 nm, as shown in Figure 2b, the superconductivity disappears completely and a gentle upturn in resistance is observed below about 10 K. Further decreasing the thickness down to 36 nm or 30 nm, the resistances maintain metallicity as the temperature decreases, then perform an unexpected abrupt increase below ~7 K followed by a resistance plateau.

**Figure 3:**
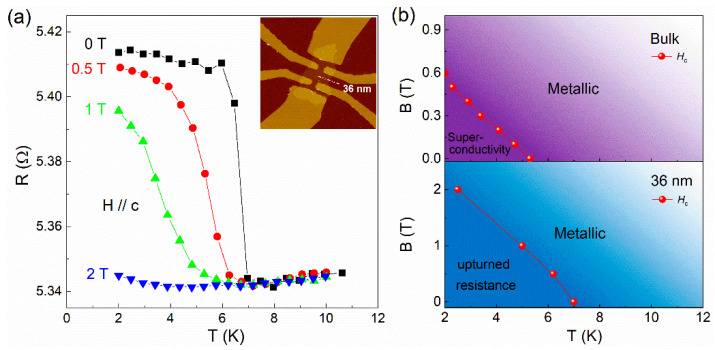
(**a**) Temperature dependence of resistance of *β*-PdBi_2_ flake under different magnetic fields along *c* axis. Inset: AFM image of a fabricated *β*-PdBi_2_ sample in a Hall-bar geometry. (**b**) The *H*_c_-*T*_c_ phase diagram of bulk β-PdBi_2_ (upper) and 36 nm nanoflake (below).

**Figure 4:**
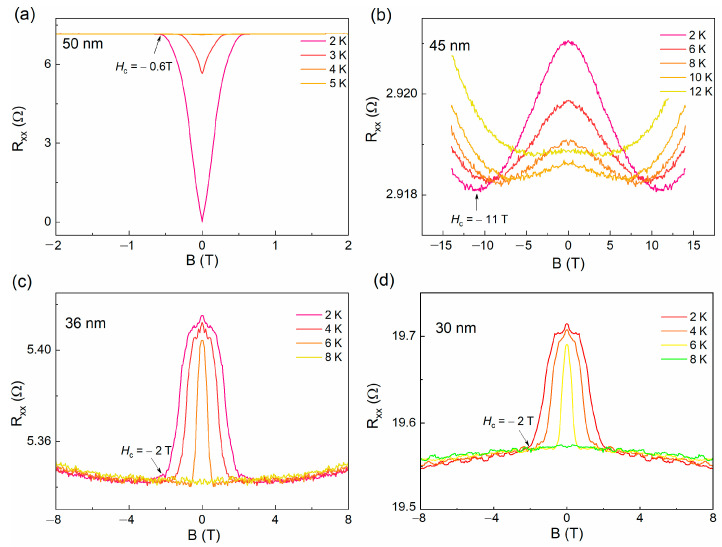
The magnetic dependence of resistance curves at different temperatures in *β*-PdBi_2_ flakes with varying thicknesses of (**a**) 50 nm, (**b**) 45 nm, (**c**) 36 nm and (**d**) 30 nm, respectively.

To understand the nature of resistance upturn observed in *β*-PdBi_2_ flakes below ~45 nm, we carried out magnetic field dependent transport measurements, as shown in Figure 3a, where the plateau was suppressed with the increase in applied magnetic field. When the field increases up to 2 T, the plateau behavior of resistance is completely suppressed. Intuitively, the plateau feature of the R-T curves in the thinner nanoflakes (*d* < 45 nm) is in opposite to a superconducting behavior, but its critical parameters, such as *T*_c_ and *H*_c_ are very similar to those observed in thick nanoflakes (*d* > 45 nm) for a superconductor, such as the *H_c_-T_c_* phase diagram, as shown in Figure 3b. In other words, the plateau feature might be relative to the superconducting nature in thinner nanoflakes, which is similar to those in superconductor films, such as 2*H*-NbSe_2_ [22,25], 2*H*-NbS_2_ [29] and Mo_x_Si_1-x_ [30]. Figure 4a displays the magnetic field-dependent resistance of 50 nm thick β-PdBi_2_ nanoflake, the superconducting feature is represented by the sharp resistance jump at a critical field *H*_c_. With a further reduction in the thickness down to below 45 nm, negative MR curves are observed, as shown in Figure 4b–d. For all samples, the negative MR survive below the onset temperatures of the resistance plateau. As the magnetic field increases, the negative MR curves present a slope change at the critical magnetic field *H*_c_.

**Figure 5:**
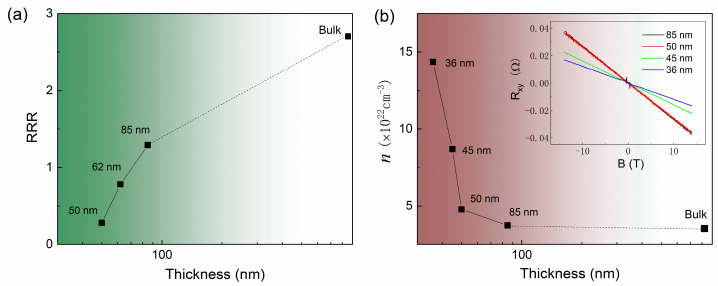
(**a**) The thickness dependence of residual resistance ratio (*RRR*) in β-PdBi_2_. (**b**) The thickness dependence of carrier concentration in *β*-PdBi_2_. The inset shows the Hall resistance of various thickness at *T* = 2 K.

Previous studies suggest that the disorder plays an important role in the transport behaviors in low dimensional system [19,29,30,31]. As the thickness decreases, the thinner samples provide more disorders in the electrically active regions, which were indicated by the reduced residual resistance ratio (*RRR*) value (*RRR* = (*R* (300 K) − *R* (*T*_c_))/*R* (*T*_c_)), as shown in Figure 5. The superconductivity will be locally suppressed due to the enhanced disordering, but survives in other areas, forming “local superconductivity” without a global long-range phase coherence [32,33,34]. In other words, the local pairing of superconductivity may survive in nanoflakes with thicknesses below 45 nm; very similar cases have been studied extensively in ultrathin 2D granular Al [35], Bi [36], In, Ga, and Pb films [37], and the superconducting LaAlO_3_/SrTiO_3_ interface [38,39]. However, we also noted that similar electronic transport behaviors were reported in high-quality Bi_2_Se_3_ thin films contacted by superconducting (In, Al, and W) electrodes [40]. The interplay between the cooper pairs of the electrodes and the spin-polarized current of the surface states in Bi_2_Se_3_ was proposed to be the possible reason. Similar to the topological insulator Bi_2_Se_3_, the topological superconductor candidate *β*-PdBi_2_ holds spin-polarized topological surface states that have been observed by ARPES experiments. However, both the theoretical calculations and the experimental results indicate that the Dirac-cone surface states have a great influence on transport properties only in ultrathin, even monolayer β-PdBi_2_ film [20,21]. The thicknesses of 30 nm to 50 nm in this work seems insufficient to induce a dominant surface state because the Dirac-cone surface is suggested to be located far away from the Fermi level when the thickness is reduced below eleven PdBi_2_ layers [10,20]. We are not sure which physical mechanism could contribute to such unusual transport phenomena; more work in single layer flakes is needed to fully understand the exotic behaviors of the topological superconductor *β*-PdBi_2_.

To get more information of the carrier, we carried out Hall resistance measurements on *β*-PdBi_2_ samples with different thicknesses at *T* = 2 K. The inset of Figure 5b displays the magnetic field dependence of Hall resistance. The completely negative slopes in the field range from −14 T to 14 T for all samples suggests that the electron-type charge carriers dominate the charge transport. The estimated carrier concentration of bulk *β*-PdBi_2_ is about 3.54 × 10^22^ cm^−3^, which is larger than previous report [41] but with the same order of magnitude. As the thickness decreases to 50 nm, the carrier concentration changes gently and is comparable to that of the bulk sample. However, with further reductions in the thickness to lower than 50 nm, where the samples present upturned resistance behavior at low temperatures, the carrier concentration increases rapidly. The abruptly increase in carrier concentration in Figure 5b indicates that the size effect may shift the Fermi level and result in a larger electronic density of states.

## 4. Summary

In this work, we perform systematic study on the evolution of superconductivity in layered topological superconductor *β*-PdBi_2_ flakes with varying thickness. We find that the *T*_c_ decreases with decreasing thickness down to about 45 nm, below which the thin *β*-PdBi_2_ nanoflakes eventually undergo an opposite behavior with a resistance abrupt jump near *T*_c_, followed by a magnetic field-dependent transport behavior. We attribute this unusual behavior to the enhanced disordering with decreasing thickness. Several possible explanations of the upturned resistance in thinner nanosheets are discussed, we expect that our research will encourage further theoretical and experimental studies on its originations.

## Data Availability

The data presented in this study are available on request from the corresponding author.
